# Profilin Isoforms Modulate Astrocytic Morphology and the Motility of Astrocytic Processes

**DOI:** 10.1371/journal.pone.0117244

**Published:** 2015-01-28

**Authors:** Stefanie K. Schweinhuber, Tania Meßerschmidt, Robert Hänsch, Martin Korte, Martin Rothkegel

**Affiliations:** 1 Cellular Neurobiology, Zoological Institute, TU Braunschweig, Braunschweig, Germany; 2 Molecular and Cell Biology of Plants, Institute of Plant Biology, TU Braunschweig, Braunschweig, Germany; University of Nebraska Medical Center, UNITED STATES

## Abstract

The morphology of astrocytic processes determines their close structural association with synapses referred to as the ‘tripartite synapse’. Concerted morphological plasticity processes at tripartite synapses are supposed to shape neuronal communication. Morphological changes in astrocytes as well as the motility of astrocytic processes require remodeling of the actin cytoskeleton. Among the regulators of fast timescale actin-based motility, the actin binding protein profilin 1 has recently been shown to control the activity-dependent outgrowth of astrocytic processes.

Here, we demonstrate that cultured murine astrocytes in addition to the ubiquitous profilin 1 also express the neuronal isoform profilin 2a. To analyze the cellular function of both profilins in astrocytes, we took advantage of a shRNA mediated isoform-specific downregulation. Interestingly, consistent with earlier results in neurons, we found redundant as well as isoform-specific functions of both profilins in modulating cellular physiology. The knockdown of either profilin 1 or profilin 2a led to a significant decrease in cell spreading of astrocytes. In contrast, solely the knockdown of profilin 2a resulted in a significantly reduced morphological complexity of astrocytes in both dissociated and slice culture astrocytes. Moreover, both isoforms proved to be crucial for forskolin-induced astrocytic stellation. Furthermore, forskolin treatment resulted in isoform-specific changes in the phosphorylation level of profilin 1 and profilin 2a, leading to a PKA-dependent phosphorylation of profilin 2a. In addition, transwell assays revealed an involvement of both isoforms in the motility of astrocytic processes, while FRAP analysis displayed an isoform-specific role of profilin 1 in the regulation of actin dynamics in peripheral astrocytic processes. Taken together, we suggest profilin isoforms to be important modulators of astrocytic morphology and motility with overlapping as well as isoform-specific functions.

## Introduction

As integral part of the tripartite synapse, astrocytes modulate neuronal excitability as well as synaptic transmission and plasticity [1]. *In vivo*, astrocytes display a complex and ramified morphology [2], which is essential for their interaction with surrounding structures like blood vessels, other glial processes and synapses. Their thin, heavily branched and highly motile structures referred to as peripheral astrocytic processes (PAPs) ensheath the pre- and postsynapse. At the synapse, astrocytes perform a variety of important functions to support synaptic transmission such as the re-uptake of glutamate [3], the co-activation of N-methyl-d-aspartate (NMDA) receptor by D-serine [4], and the modification of synaptic strength by astrocyte-derived glutamate [5]. Astrocytes react to neuronal stimuli with morphological plasticity: for instance, the coverage of the synapse by the corresponding PAP is increased upon whisker stimulation *in vivo* [6]. Moreover, retraction of PAPs from the synapse results in alteration of synaptic transmission [3]. The highly dynamic morphology of PAPs depends to a great extent on the actin cytoskeleton, as application of cytochalasin D drastically impairs the motility of astrocytic processes [7]. The microfilament system is, unquestionably, also responsible for performing larger scale morphological changes in astrocytes [8]. The role of morphological plasticity of astrocytes and its underlying mechanisms are commonly addressed using dissociated cultures of astrocytes. In contrast to the heavily branched morphology *in vivo*, astrocytes in culture attain a flat, epithelioid morphology. Still, they were shown to at least partially reacquire the *in situ* phenotype by co-cultivation with dissociated neurons [9] or, in an actin-dependent manner, after the application of dibutyryl cAMP (db-cAMP, [8]). This process, often referred to as stellation, is characterized by cytoplasmic retraction, the formation of thin processes and actin remodeling [10–12]. Actin dynamics are tightly regulated by a plethora of actin binding molecules. Prominent regulators of actin dynamics are profilins (PFN), which are involved in a variety of cellular functions. Profilins bind to monomeric actin in a 1:1-complex and facilitate the exchange of ADP to ATP at actin monomers and are therefore involved in actin treadmilling and the elongation of new filaments [13]. In animal cells, this process is under the regulation of numerous large, multidomain proteins of the Ena/VASP and formin families that can bind actin, profilin and profilin:actin complexes and are crucial members of various signalling pathways [14,15]. In addition to binding to actin and the polyproline stretches of formins and Ena/Vasp and other poly-proline proteins, profilins can bind to acidic phospholipids some of which are also involved in signal transduction [13,15,16]. Hence, profilins are thought to be engaged in linking signal transduction to actin filament formation, and in accordance with this view, they were found to localize with actin, VASP, WAVE and formins in membrane-apposed, dynamic regions of cells (for original references, see [13,14]. However, the wealth of different actin-based motility processes in cells, like locomotion, membrane trafficking, cytokinesis, morphogenesis of cellular protrusions and adhesion complexes, probably requires regional modulation of profilin-ligand interactions. Thus, different phospholipids, different formins or Ena/VASP proteins, different actin isoforms and different profilins may specialize in various motility processes. Consistent with such a concept is the notion that profilins occur in a number of isoforms which in part show tissue-specific expression. The number, structural relationship and evolutionary origin of profilin isoforms from vertebrates has been investigated in detail [17], but their biochemical and cell biological properties has been analyzed only in a restricted number of cases. In mammals, four discrete profilin genes, one of which is spliced in two ways, give rise to five different isoforms. Profilin 1 (PFN1) is ubiquitously expressed and is apparently engaged in general motility functions, like cell migration, cytokinesis and adhesion in all cell types (cf. [13]. This concept is supported by the finding that loss of PFN1 in knock out mice is lethal very early in development [18]. Profilin 2a (PFN2a), the major splice form of profilin 2, is primarily expressed in the brain, while a minor splice product, PFN2b, was identified in the kidney [19,20]. Furthermore, PFN3 and PFN4 were identified in mammalian testis [21,22], where PFN4 seems specifically involved in the maturation of spermatids [22]. Recent studies on the role of profilins in the CNS have revealed isoform-specific functions of PFN1 and PFN2a in neuronal cells [23–27]. The PFN2a-specific knockdown has a significant effect on the morphology of CA1 pyramidal neurons [28]. Recently, PFN1 has been shown to regulate the Ca^2+^ triggered outgrowth of PAPs in dissociated astrocytes [29]. Furthermore, PFN1 has been implicated as a regulator of process length in Bergmann glia [30] and is required for glial cell adhesion and radial migration of cerebellar granule neurons [30]. Thus, the precise roles of different profilin isoforms in astrocytic functions remains elusive. Here, we show the expression of two profilin isoforms in cultured astrocytes and describe both overlapping as well as isoform-specific functions of PFN1 and PFN2a in this context. Particularly, we identify both profilin isoforms as regulators of dynamic changes in astrocytic morphology, but only PFN2a as being important for the maintenance of the astrocyte specific stellate shape. In addition, we use fluorescence recovery after photobleaching (FRAP) to demonstrate that actin dynamics in astrocytes are solely modulated by PFN1. Taken together our findings demonstrate both overlapping and isoform-specific cellular functions of both profilin isoforms expressed in astrocytes.

## Results

### Cultured astrocytes express PFN1 and PFN2a

**Fig 1:**
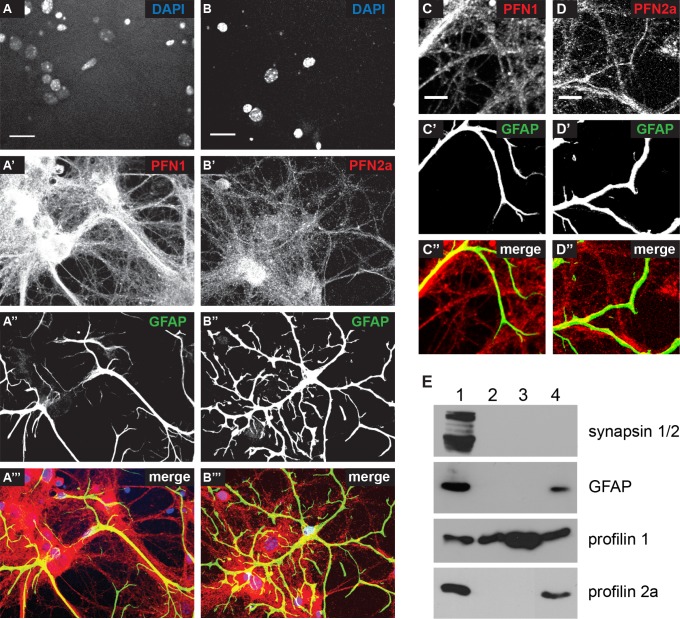
Astrocytes express two profilin isoforms. (A, B) Dissociated hippocampal co-cultures of astrocytes and neurons. Both astrocytes and neurons express (A) PFN1 and (B) PFN2a. Scale bar: 20 μm (C, D) Detail images of astrocytic filaments identified by GFAP staining, expressing (C) PFN1 and (D) PFN2a. Scale bar: 10 μm (E) Western blot analysis of cultured astrocytes (4) compared to adult mouse brain (1), spleen (3) and liver (2). Astrocytic cultures as well as brain lysate show clear immunoreactivity to a PFN2a specific antibody, whereas the liver and spleen lysates do not show a signal. Immunoreactivity to the ubiquitous profilin isoform PFN1 is obvious in all probed cells and tissues.

Although the profilin isoforms display a highly conserved three dimensional structure, they interact with different ligands [13,31] and are thereby thought to be involved in different cellular functions [24,26,32,33]. Accordingly, we investigated here the isoform-specific cellular role of profilin 1 and 2a in astrocytes. First, we tested whether astrocytes express PFN1 as well as PFN2a, as it is the case for neurons. Therefore, we used primary hippocampal cultures containing both neurons and astrocytes. In this context, GFAP positive cells were also stained with a PFN2a-specific antibody (Fig. 1A-D). In addition, the expression of PFN1 and PFN2a in astrocytes was analyzed by immunoblotting with isoform-specific antibodies in dissociated astrocytic cultures devoid of neurons and, as controls in total extracts of mouse brain, liver and spleen (Fig. 1E). Whole brain extracts as well as glia culture extracts were immunoreactive to both PFN1 and PFN2a specific antibodies, whereas the mouse liver and spleen were only positive for PFN1.

### Profilins are important for spreading of astrocytes

**Fig 2:**
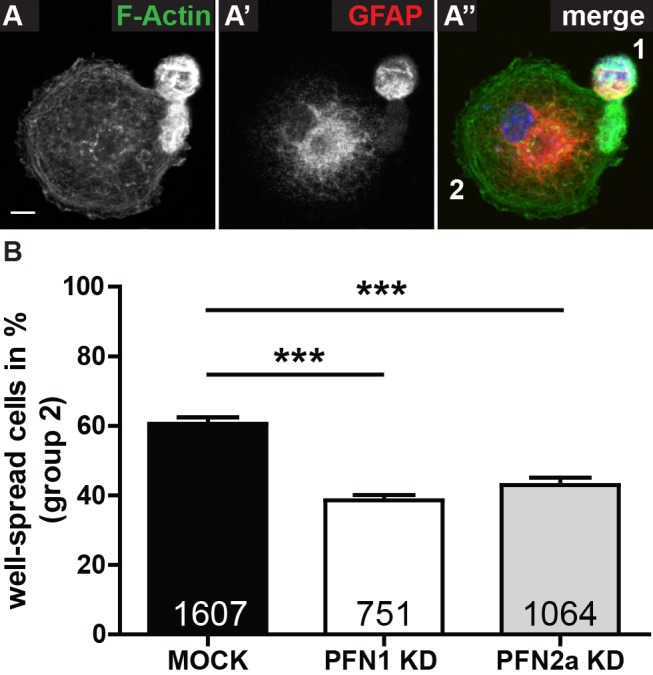
Both profilins have a drastic effect on spreading of astrocytes. (A) Spreading assay. Astrocytes were allowed to adhere to PLO-coated coverslips for 6 hours, were subsequently fixed and stained with (A) phalloidin as well as (A’) anti-GFAP to identify astrocytes and DAPI to stain for nuclei (A”). According to their morphology and the appearance of their actin cytoskeleton, cells were classified into two groups: round cells lacking obvious spreading (group 1) and well spread cells (group 2). Scale bar: 5 μm (B) Statistical analysis of astrocytes transduced with either Usifluc (MOCK, black), Ush1.3 (PFN1 KD, white) or Ush2.13 (PFN2a KD, light grey) subjected to the spreading assay in A and subsequently classified into groups 1 and 2. 60.6±1.9% of control cells were classified into group 2, whereas only 38.6±1.5% of PFN1 knockdown cells and 43.0±2.1% of PFN2a knockdown cells were well-spread according to criteria. Quantitative data obtained from 3 independent experiments was tested for significance by one-way ANOVA followed by a *post-hoc* Tukey’s Multiple Comparison Test. Significance is indicated as follows *p<0.05; **p<0.01; ***p<0.001. Data are shown as mean ± SEM.

Cell adhesion and cell spreading are closely related to actin dynamics and are known to be regulated by profilin isoforms in avian fibroblasts [32]. Therefore, we analyzed the consequences of profilin isoform-specific downregulation in astrocytes on their spreading. For our studies we took advantage of a shRNA mediated, isoform-specific knockdown approach by the use of a lentiviral vector targeting isoform-specific profilin mRNA and expressing a membrane-bound GFP (eGFP-f) as reporter (S1 Fig.). Astrocytic cultures were transduced with lentiviral particles encoding the appropriate shRNA, subsequently seeded onto cover slips and the attached cells were analyzed by fluorescence microscopy. In accordance to cell morphology and microfilament organization, the attached cells were classified into round cells lacking obvious spreading (Fig. 2A, group 1) and well-spread cells (Fig. 2A, group 2). Indeed, statistical analysis of well-spread astrocytes (Fig. 2B) revealed a highly significant decrease upon knockdown of either profilin isoform (Fig. 2B, white and grey) to about three-quarter of control level (Fig. 2B, black, 60.6±1.9%). Whereas no significant differences between astrocytes expressing PFN1 (white, 38.6±1.5%) or PFN2a (light grey, 43.0±2.1%) specific shRNAs were observed. These findings suggested both profilin isoforms as regulators of astrocytic spreading.

### Knockdown of profilins alters morphology of astrocytes

**Fig 3:**
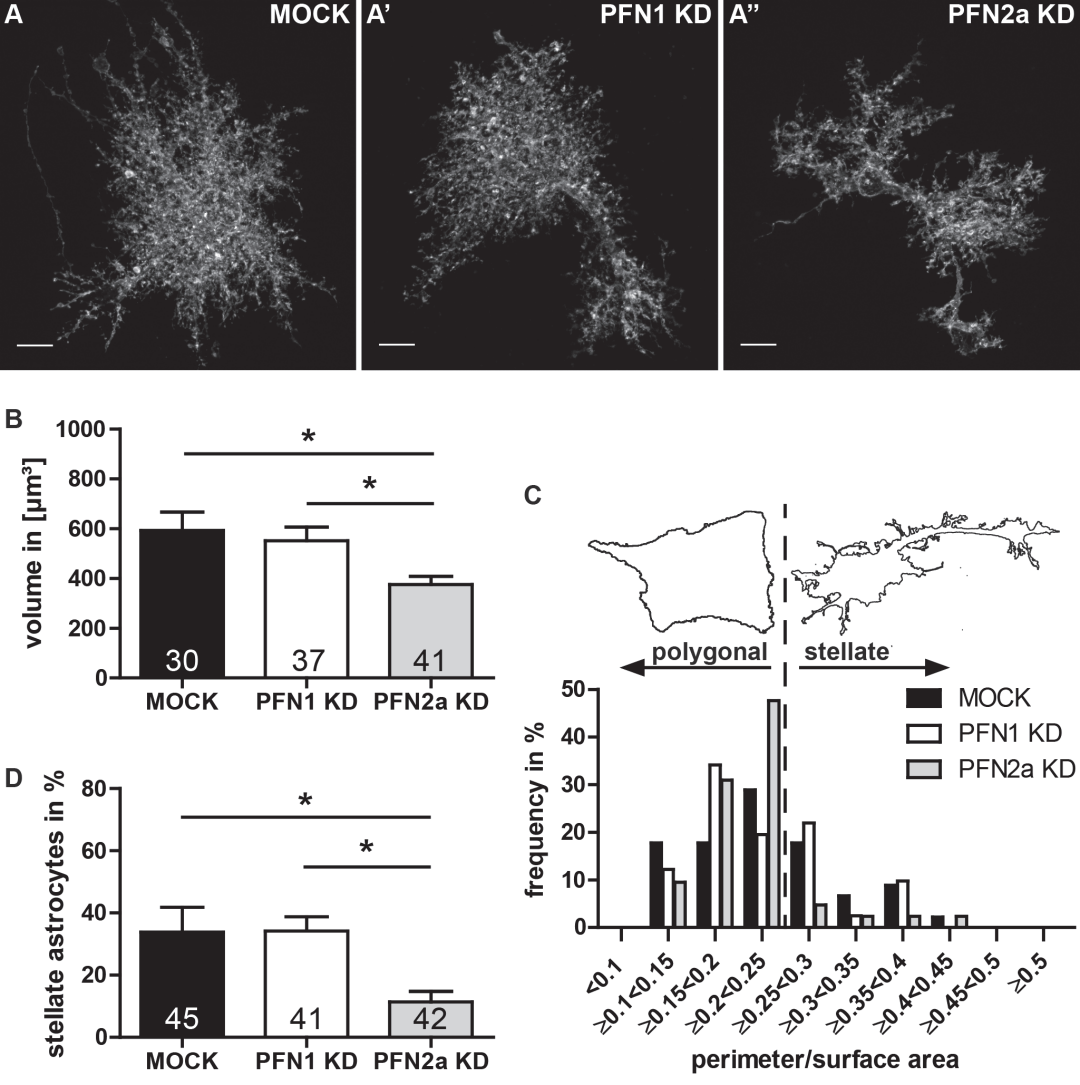
PFN2a modulates morphology of astrocytes in organotypic slices and dissociated cultures. (A-A”) Morphology of astrocytes in cortical organotypic slice cultures is similar to the *in vivo* appearance. Images of representative astrocytes (A) MOCK transfected, expressing a PFN1 specific shRNA (A’) and a PFN2a specific shRNA (A”), respectively. Scale bar: 10 μm. (B) Statistical analysis of the volume of astrocytes from cortical slice cultures expressing the shRNA specific for PFN2a (375.6±33.2 μm²) displayed a significant reduction of their volume compared to control (592.6±74.3 μm²) or PFN1 knockdown (551.8±54.4 μm²) astrocytes. (C) Categorization of astrocytes from dissociated cultures into 2 groups: stellate and polygonal. Astrocytes were subdivided into different classes according to their ratio between cell perimeter and cell area. Subsequently, cells with a ratio above 0.25 were added up to achieve the percentage of stellate astrocytes, the remaining were totaled to the group of polygonal astrocytes. The border between these two groups is indicated as dashed line. (D) The amount of stellate cells in dissociated cultures is significantly reduced in the group of PFN2a reduced cells. Quantitative data obtained from 3 independent experiments was tested for significance by one-way ANOVA followed by a *post-hoc* Tukey’s Multiple Comparison Test. Significance is indicated as follows *p<0.05; **p<0.01; ***p<0.001. Data are shown as mean ± SEM.

Recent studies revealed PFN2a as a modulator of the morphology in pyramidal hippocampal neurons [28], while in Bergmann glia the loss of PFN1 led to a reduced process length [30]. Consequently, we addressed the question whether profilins modulate astrocytic morphology in an isoform-specific manner. Initially, the volume of protoplasmic astrocytes in cortical slice cultures transfected by biolistic delivery of the appropriate shRNA encoding plasmids (S1 Fig.) pRNAT-siFluc (MOCK), pRNAT-1.3 (PFN1 KD) or pRNAT-2.13 (PFN2a KD), respectively, was analyzed (Fig. 3A-A”, 3B). In comparison to control cells (592.6±74.3 μm²; Fig. 3B, MOCK, black) the knockdown of PFN2a resulted in a significantly reduced volume (375.6±33.2 μm²; Fig. 3B, grey) while the volume of PFN1 knockdown astrocytes was not altered (551.8±54.4 μm²; Fig. 3B, white). This experiment proved the relevance of PFN2a expression in astrocytes. In a second set of experiments on the physiological role of profilins, we used dissociated primary astrocytes as a well-established cell system in order to analyze morphological plasticity. To reduce the endogenous profilin levels, primary astrocytes were transfected with plasmids encoding the appropriate shRNA and their morphology was analyzed by fluorescence microscopy. To this aim, we determined cell perimeter and cell surface area of the transfected cells with FIJI software. Downregulation of either PFN1 or PFN2a resulted in a significantly reduced cell perimeter and cell surface area compared to mock transfected cells (S2 Fig.). This suggests both profilins as regulators of cell size. We than classified the transfected astrocytes by the ratio between their cell perimeter and their cell area in a relative frequency distribution analysis. Thereby, the cell shape is corrected for the reduction in cell size caused by the loss of profilin isoforms. The resulting frequency analysis (Fig. 3C) was used to subdivide the cells in two groups [34]. The majority of cells at basal conditions in serum containing media showed a perimeter to area ratio below 0.25, referred to as polygonal. Astrocytes with a perimeter to area ratio above 0.25 were classified as stellate (Fig. 3C). Applying this classification on the transfected astrocytes revealed, that the group of cells expressing the PFN2a specific shRNA contained significantly less stellate cells (Fig. 3D; 11.4±3.4%) compared to the two other groups (MOCK: 33.9±7.9%; PFN1 KD: 34.2±4.6%). Although our results indicate both profilin isoforms as modulators of cell size, only PFN2a might function as a regulator of morphological complexity.

### Profilins are essential for forskolin induced stellation

Increased cAMP levels lead to actin and Arp2/3 complex-dependent dynamic changes in the cytoskeleton [8,34] of astrocytes. Therefore, we induced remodeling of the astrocytic microfilament system by forskolin, a stimulation agent raising intracellular cAMP levels, to determine whether profilin isoforms play a role in actin- dependent changes of the astrocytic morphology.

**Fig 4:**
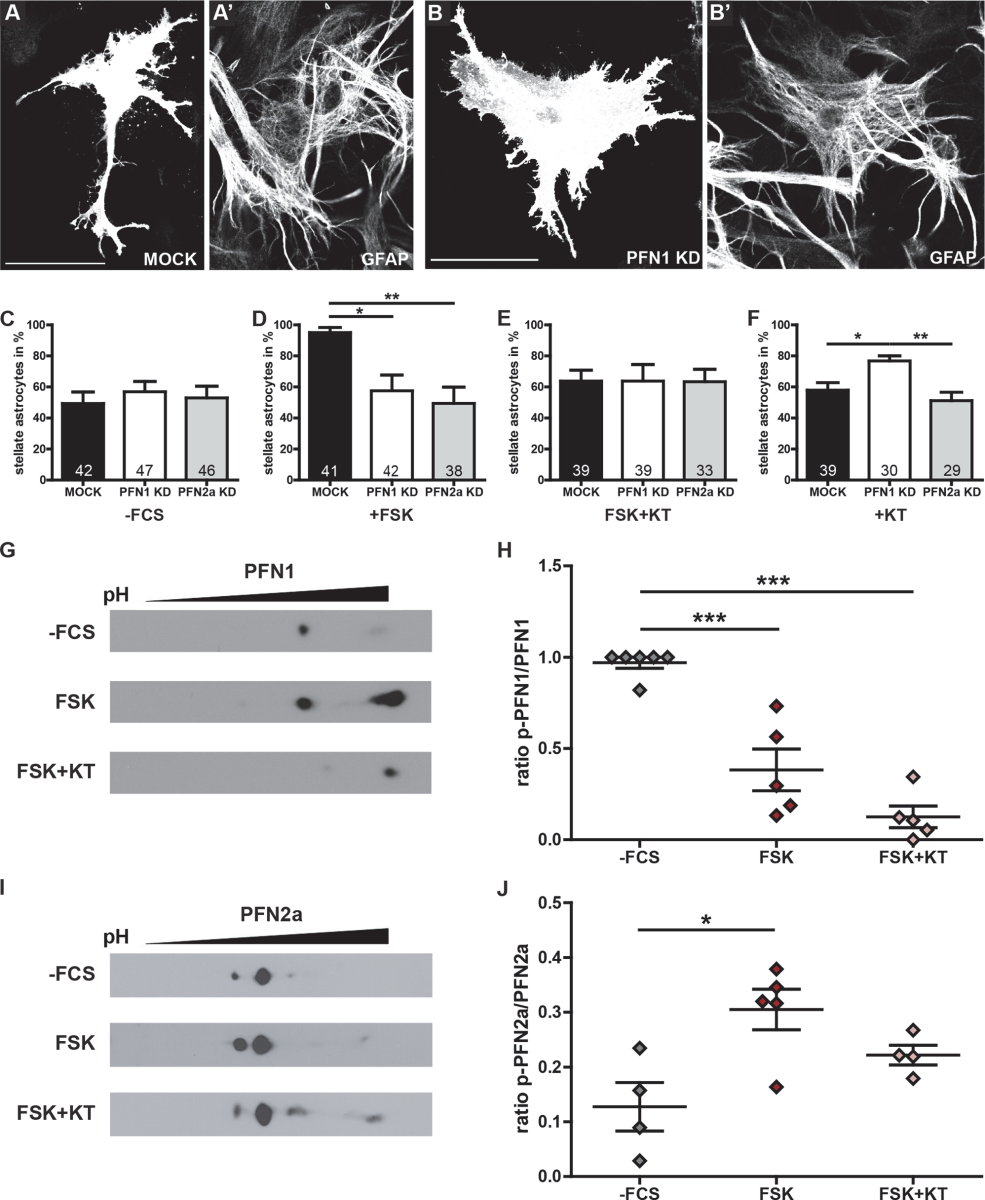
PKA-dependent stellation induces changes in phosphorylation of both profilins. (A) MOCK transfected astrocyte attains a stellate phenotype upon stimulation with 10 μM forskolin (FSK) for 2 hours. (B) Astrocyte expressing a shRNA specific for PFN1 treated with forskolin. The fluorochrome expressing cell does not contract properly. (C, D, E, F) Astrocytes transfected with either MOCK, PFN1 KD or PFN2a KD were categorized into stellate and polygonal cells. (C) 4 hours serum starved astrocytes showed no alterations comparing the 3 different groups (MOCK 49.3±7.4%; PFN1 KD 56.9±6.6%; PFN2a KD 52.9±7.6%) and served as control condition. (D) MOCK transfected astrocytes are significantly more stellate upon forskolin treatment (95.0±3.3%) compared to both PFN1 KD (57.5±10.1%) or PFN2a KD cells (49.4±10.5%). (E) Simultaneous co-application of FSK and the PKA inhibitor KT5720 (KT, 100 nM) abolished the stellation effect of FSK on MOCK transfected cells (MOCK 63.8±7.0%; PFN1 KD 63.8±10.7%; PFN2a KD 63.3±8.0%). (F) Upon PKA inhibition by KT5720 treatment, the downregulation of PFN1 (76.7±3.3%) leads to a significant increase in stellate astrocytes relative to MOCK (57.9±4.8%) or PFN2a knockdown (51.2±5.4%). (G) 2D gel electrophoresis with subsequent Western Blot analysis of PFN1. Astrocytic cultures were kept in DMEM without supplement (-FCS), treated with 10 μM forskolin for 2 hours (FSK) or treated with 10 μM forskolin for 2 hours in the presence of 100 nM KT5720 (FSK+KT). (H) Quantification of the phosphorylation status of PFN1. Dot intensities were measured with Image J. Intensity values of all dots were totaled and the intensity of the left dot was divided through the total intensity. The mean relative amount of phosphorylated PFN1 was significantly decreased in forskolin treated cultures (0.38±0.11) compared to control cultures (0.97±0.03). Cultures treated with both FSK and KT attained a fraction of 0.13±0.06, which is significant to the control group but not to FSK treated cultures. (I) 2D gel electrophoresis with subsequent Western Blot analysis of PFN2a. Cultures were treated as indicated in G. (J) Quantification of the phosphorylation status for PFN2a as described in H. The mean relative amount of phosphorylated PFN2a was significantly increased in forskolin treated cultures (0.31±0.04) compared to control cultures (0.13±0.04). Cultures treated with both FSK and KT attained a fraction of 0.22±0.02, which is a non-significant decrease (p = 0.11) compared to forskolin treated cultures. Quantitative data obtained from 3 (F, H) or 4 (C-E, J) independent experiments was tested for significance by one-way ANOVA followed by a *post-hoc* Tukey’s Multiple Comparison Test. Significance is indicated as follows *p<0.05; **p<0.01; ***p<0.001. Data are shown as mean ± SEM.

Cultured astrocytes transfected with either pRNAT-siFluc (MOCK), pRNAT-1.3 (PFN1 KD) or pRNAT-2.13 (PFN2a KD) were serum starved, subsequently stimulated with 10 μM forskolin in DMEM without FCS and the induced morphological changes were compared to the appropriate untreated control (Fig. 4). Whereas mock transfected astrocytes underwent a strong contraction and thereby reduction of their cell area (Fig. 4A), the astrocytes expressing shRNAs specific for PFN1 or PFN2a, respectively, fail to contract significantly upon treatment (Fig. 4B). Quantification for the mock transfected astrocytes treated with forskolin (Fig. 4D, black) displayed the expected increase in the percentage of stellate cells (95.0±3.3%) compared to untreated controls (49.3±7.4%; Fig. 4C, black). Remarkably, neither astrocytes expressing the shRNA specific for PFN1 nor PFN2a showed a significant response to forskolin treatment (Fig. 4D; white, 57.5±10.1%; grey, 49.4±10.5%). The lack of forskolin-induced morphological changes in astrocytes expressing shRNA specific for PFN1 or PFN2a reveals a key role for both profilin isoforms in actin-dependent astrocytic stellation and demonstrates the importance of both profilins for the stellation process.

### PKA inactivation results in altered morphology of astrocytes with reduced PFN1 level

As the increase of cAMP levels directly activates the cAMP-dependent protein kinase A (PKA), we performed experiments with the co-application of forskolin and the specific PKA inhibitor KT5720 [35] to verify the role of PKA in this context. Indeed, mock transfected cells (Fig. 4E, black) treated with both FSK and KT5720 exhibit a significantly reduced percentage of stellate cells (64.6±7.0%) compared to forskolin treatment alone (Fig. 4D, black; 95.0±3.3%). Whereas in comparison to untreated controls (Fig. 4C) the mock transfected astrocytes as well as both astrocytic cultures with a reduced level of either PFN1 or PFN2a displayed a non-significant alteration in percentage of stellate astrocytes upon co-application of FSK and KT5720 (Fig. 4C, 4E, white; 54.4±5.8% to 66.3±10.0%; grey; 50.6±7.8 to 64.4±8.6%). In contrast, the application of KT5720 alone led, at least on cells with a reduced amount of PFN1, to morphological alterations (Fig. 4F). Statistical analysis of mock transfected astrocytes (Fig. 4F, black) compared to astrocytes expressing either a shRNA specific for PFN1 (PFN1 KD, white) or PFN2a (PFN2a KD, grey) revealed significant differences in their morphology. Particularly, downregulation of PFN1 resulted in a significantly increased percentage of astrocytes attaining a stellate morphology upon KT5720 treatment (76.7±3.3%) compared to both mock transfected (57.9±4.8%) and PFN2a knockdown astrocytes (51.2±5.4%). These findings led to further examination of the interplay between PKA activation or inactivation and profilins.

### Forskolin treatment modulates the phosphorylation level of profilin 1 and 2a

Since profilins were described as phosphoproteins [36,37] we analyzed the phosphorylation state of both isoforms in forskolin-treated astrocytic cultures. Therefor total extracts of astrocytes treated with either forskolin or forskolin plus KT5720 were analyzed by 2D gel electrophoresis (Fig. 4G, 4I). Forskolin treatment of astrocytic cultures led to a significant decrease in the ratio of phosphorylated PFN1 (0.38±0.11) compared to control cultures (0.97±0.03; Fig. 4H). Additionally, co-application of forskolin and KT5720 resulted in a further non-significant reduction of phosphorylated PFN1 (0.13±0.06). In contrast, 2D gel analysis of profilin 2a (Fig. 4I) revealed a significant increase in the ratio of phosphorylated PFN2a (0.31±0.04) in cultures subjected to forskolin compared to untreated controls (0.13±0.04; Fig. 4J). Additionally, co-application of forskolin and KT5720 resulted in a tendency towards a reduced ratio of phosphorylated PFN2a in astrocytic cultures when compared to merely forskolin-treated cultures (0.22±0.02, p = 0.11; Fig. 4I, 4J). These results suggested PFN2a as a possible phosphorylation target downstream of PKA and pointed to a correlation between morphological plasticity in astrocytes and profilin isoform-specific phosphorylation.

### Localization of profilins is altered by PKA activation or inactivation

**Fig 5:**
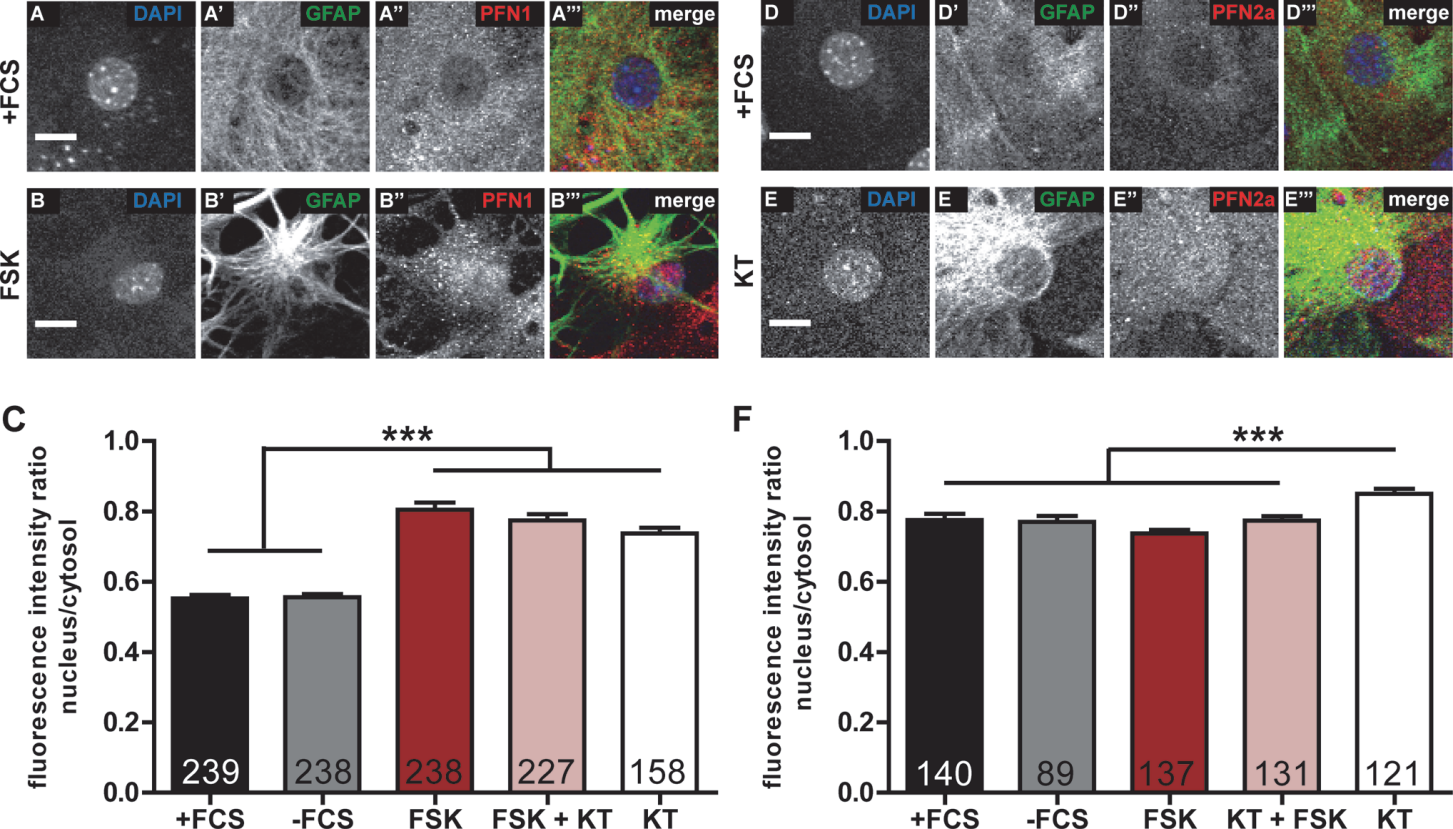
Subcellular localization of profilin isoforms is altered by forskolin treatment and PKA inhibition. (A) Control cell (+FCS) and (B) a forskolin treated cell (FSK). In addition to the morphological change, the amount of PFN1 in the nucleus of the cell treated with forskolin was increased. (C) Quantification of the PFN1 ratio between cytosol and nucleus. Treatment with either forskolin or KT5720 or both leads to an increased amount of PFN1 in the nucleoplasma of astrocytes. (D) Control cell (+FCS) and (E) a KT5720 treated cell (KT). The amount of PFN2a in the nucleus of the astrocyte is increased in comparison to control upon KT5720 treatment. (F) Quantification of the PFN2a ratio between cytosol and nucleus. Solely treatment with KT5720 increases the amount of PFN2a in the nucleoplasma. Quantitative data obtained from 3 (KT treatment) or 4 independent experiments was tested for significance by one-way ANOVA followed by a *post-hoc* Tukey’s Multiple Comparison Test. Significance is indicated as follows *p<0.05; **p<0.01; ***p<0.001. Data are shown as mean ± SEM.

PFN1 has been shown to localize both in the cytosol and in the nuclei of different mammalian cell types [27,38–41]. Also PFN2a has been described to localize in the cytosol as well as in the nuclei of neurons [27]. Therefore, we stained dissociated astrocytic cultures with isoform-specific profilin antibodies and analyzed the subcellular distribution of the two profilins in these cells. While under basal conditions (Fig. 5A) PFN1 is predominantly localized to the cytosol and the nucleus appears almost devoid of fluorescence, the fluorescence intensity in the nucleus increased upon application of forskolin (Fig. 5B). To quantify the subcellular localization, we measured the fluorescence intensities in the nucleus as well as in a cytosolic part around the nucleus and compared the ratio between these two (Fig. 5C). No differences could be observed in the fluorescence intensity ratios of controls with and without FCS. The treatment with either forskolin or KT5720 as well as with both reagents however, led to a highly significant increase of the amount of PFN1 in the nucleus. These results pointed to a PKA-independent translocation of PFN1 upon forskolin-stimulated astrocytic stellation. In addition, we analyzed the subcellular localization of PFN2a in astrocytes. Under control conditions (Fig. 5D) PFN2a is rather localized in the astrocytic cytosol, but, in contrast to PFN1, no forskolin induced alterations could be detected (Fig. 5F). Strikingly, PFN2a specifically translocated to the nucleus upon KT5720 treatment (Fig. 5E). The quantification of the fluorescence intensity ratios exhibited no alterations for serum containing, serum starved, forskolin-treated as well as forskolin plus KT5720 treated cells (Fig. 5F). Solely treatment with KT5720 led to a highly significant increase in PFN2a fluorescence intensity in the nuclei of analyzed cells compared to all other conditions. These findings suggested a regulation of PFN2a localization downstream of PKA.

### Isoform-specific roles of profilins in regulating actin-dependent motility of astrocytic processes

**Fig 6:**
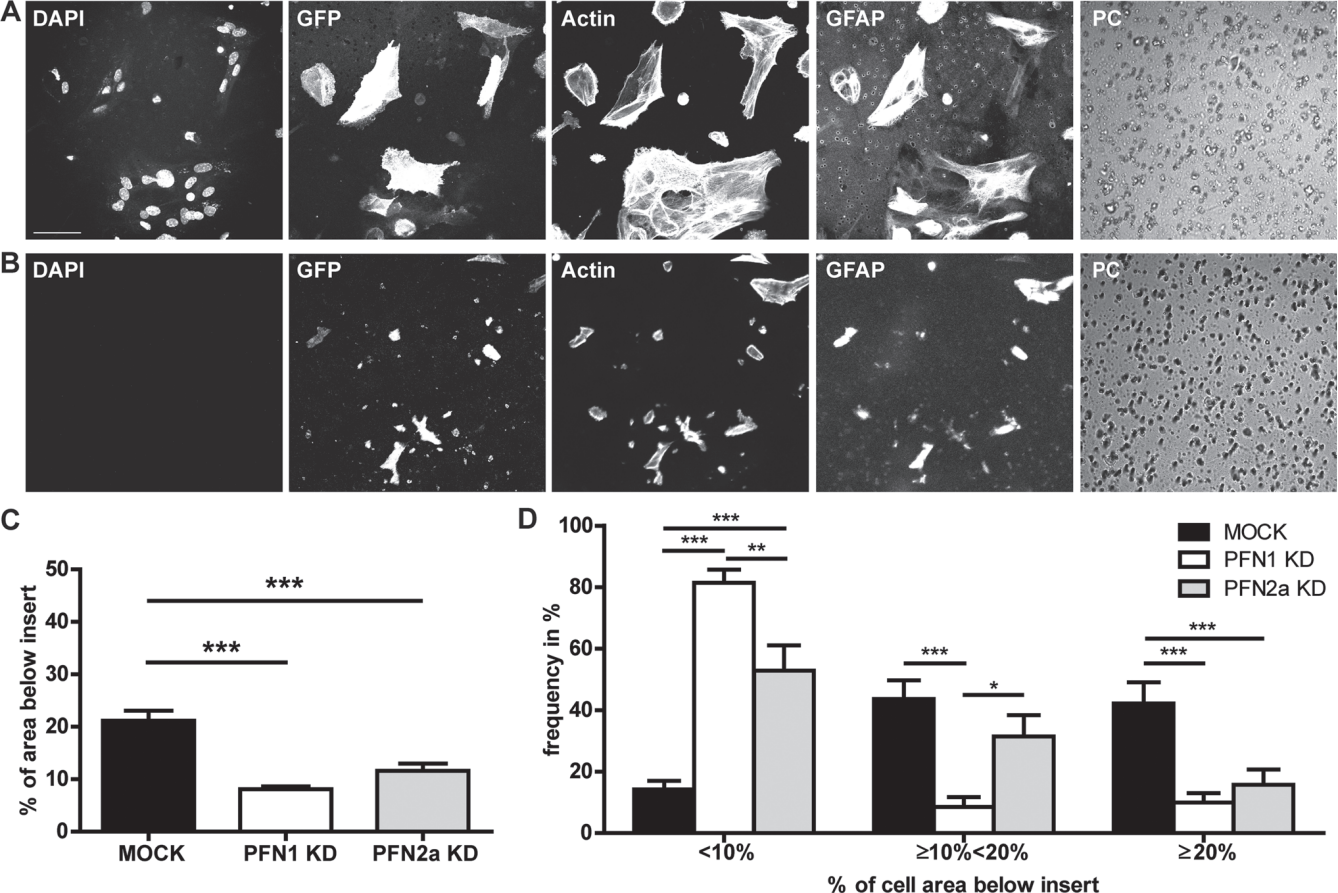
Downregulation of profilins impairs the motility of astrocytic processes. (A) Upside and (B) bottom side of a transwell insert after 24 hours of incubation. Cells were stained for GFAP to identify astrocytes. DAPI positive nuclei are solely visible on the upside of the membrane. Scale bar: 50 μm (C) Quantification of GFP and GFAP positive membrane on the bottom side in relation to the upper side. Reduction of either profilin isoform resulted in a significant decrease of GFP positive membrane on the lower surface of the insert. At least, 300 cells were analyzed for each group. (D) For a more detailed analysis, results were subdivided into three classes depending on the percentage of the membrane ratio from upper surface to lower surface of the inserts. Samples grouped into the <10% class displayed only a sparse pattern of processes on the bottom side. Significant more samples with a reduced amount of PFN1 compared to controls and PFN2a knockdown were classified into this group. Quantitative data obtained from 4 independent experiments was tested for significance by either one-way ANOVA or two-way ANOVA (D) followed by a *post-hoc* Tukey’s Multiple Comparison Test. Significance is indicated as follows *p<0.05; **p<0.01; ***p<0.001. Data are shown as mean ± SEM.

The cellular plasticity of astrocytes is directly linked to the motility of their processes, which in turn depends on a dynamic actin cytoskeleton [7]. Furthermore, the importance of PFN1 to calcium induced outgrowth of astrocytic processes was recently demonstrated [29]. Here, we investigated whether both profilin isoforms are involved in regulating the motility of astrocytic processes. For that purpose, we took advantage of a Boyden-chamber based transwell assay [42,43]. After 24 h of incubation, while the majority of the cell membrane was still localized to the upper side of the Boyden chamber membrane (Fig. 6A), cell processes were also visible at the bottom side (Fig. 6B). The extension of cellular processes was independent of cell body translocation, as there were no nuclei visible at the bottom side of the membrane (Fig. 6B, DAPI). Processes extending through the pores contained filamentous actin (Fig. 6B, actin) suggesting that their motility might be based on actin dynamics. Quantification of the transwell assay (Fig. 6C) revealed for controls (MOCK, black) 21.1±6.1% of total cell area to be located on the bottom side. Downregulation of either profilin isoform significantly decreased the percentage of cell area on the bottom site to 8.1±1.7% for PFN1 (Fig. 6C, white) and 11.6±4.5% for PFN2a (Fig. 6C, grey), respectively. To further characterize the effects of the reduction of PFN1 or PFN2a protein levels on the motility of astrocytic processes, we classified the fields of view into groups depending on their proportion of cell membrane on the bottom side (Fig. 6D). In about 80% of analyzed fields of view, the knockdown of PFN1 led to a significant reduction (bin “<10%”) of cell area on the bottom side in comparison to PFN2a KD (52.86±8.3%) and mock transfected cultures (14.2±2.8%). Contrarily, in about 31% percent of analyzed fields of view the knockdown of PFN2a only led to a moderate declined cell area on the bottom side (bin “≥10% <20%”), which is significant to PFN1 knockdown cultures (8.6±3.2%) but not significantly altered compared to controls (43.6±6.1%). These findings suggest a major role of PFN1 in the serum induced motility of astrocytic processes.

**Fig 7:**
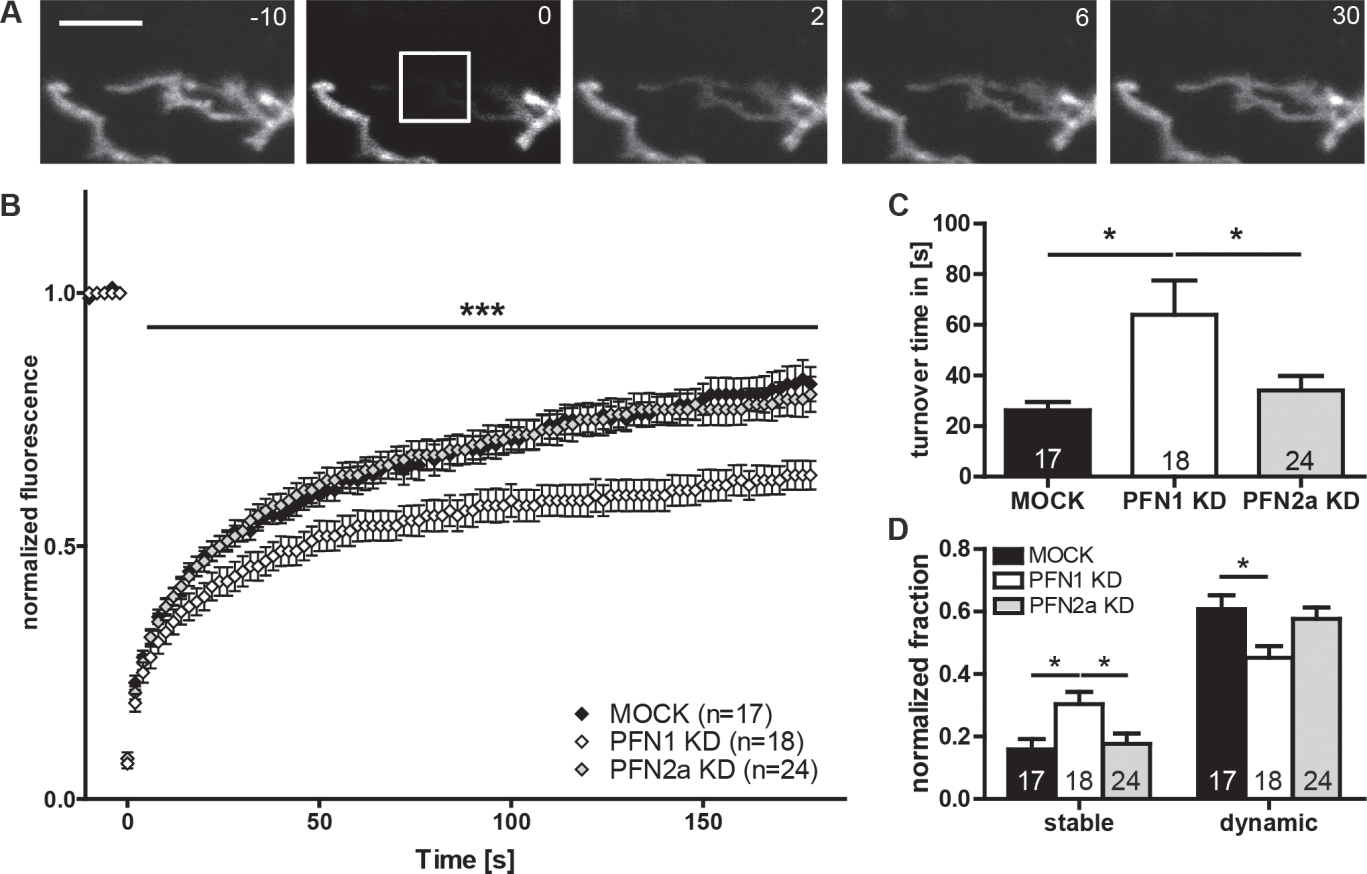
FRAP analysis reveals PFN1 as major regulator of actin dynamics in astrocytic processes. (A) Astrocytes co-expressing eGFP-actin and siFluc, shPFN1 or shPFN2a, respectively, were used for FRAP analysis to determine the effect of profilins on actin dynamics in PAPs. eGFP-actin fluorescence was bleached from a square of 4x4 μm (white rectangle) containing one or two small astrocytic processes and fluorescence recovery was monitored for 180 s. Scale bar: 5 μm. (B) Recovery curve for control, as well as PFN1 and PFN2a knockdown astrocytes. Solely intensity values of cells with a reduced level of PFN1 are significantly lower compared to control. (C) Turnover time is accordingly increased for cells with decreased amounts of PFN1 (63.89±13.61 s) compared to MOCK (26.24±3.3 s) and PFN2a KD (34.08±5.7 s). (D) Analysis of the actin pools reveals, solely for PFN1 knockdown cells, a decrease in the dynamic and a corresponding increase in the stable pool. Quantitative data obtained from 4 independent experiments was tested for significance by either one-way ANOVA followed by a *post-hoc* Tukey’s multiple comparison test or two-way ANOVA (B). Significance is indicated as follows *p<0.05; **p<0.01; ***p<0.001. Data are shown as mean ± SEM.

By fluorescence recovery after photobleaching (FRAP) we analyzed the impact of profilin isoforms onto actin dynamics within astrocytic processes. Therefore, cultured astrocytes were co-transfected with a GFP-β-actin encoding plasmid and either PFN1 or PFN2a specific shRNA. Time lapse images of GFP-β-actin expressing astrocytic processes (Fig. 7A) bleached at time point 0 were taken and fluorescence recovery was monitored for a total of 3 min. Analyses of fluorescence recovery of mock transfected cells, PFN1 knockdown and PFN2a knockdown astrocytes (Fig. 7B) presented no changes in the recovery curve for astrocytes with a reduced amount of PFN2a in comparison to control (MOCK). In contrast, the decrease of PFN1 resulted in significantly lower fluorescence values. Furthermore, the actin turnover in astrocytes expressing the PFN1-specific shRNA (63.89±13.61 s) was significantly decelerated compared to mock transfected (26.24±3.3 s) and PFN2a knockdown cells (34.08±5.7 s) (Fig. 7C). These results indicated PFN1 as a regulator of actin dynamics and underline an isoform-specific role in the morphological plasticity of astrocytic processes. Additionally, based on the assumption of the existence of two pools of actin filaments differing in their stability, recovery time fluorescence curves were used to identify these different actin pools [44]: the dynamic F-actin pool as the one recovering during the observation period and the stable F-actin pool as the one which is stably associated during the experiment (S3 Fig.). Quantification of the actin pools (Fig. 7D) presented a significant decrease in the dynamic actin pool of PFN1 knockdown astrocytes as well as a corresponding increase in the stable actin pool. Once more, astrocytes lacking profilin isoform 2a showed no significant alterations compared to control astrocytes. These findings point to an isoform-specific role of PFN1 in regulating actin dynamics within astrocytic processes.

## Discussion

The two profilin isoforms PFN1 and PFN2a have been demonstrated to be differentially involved in regulating various important cellular processes in neurons [23–28,33]. The structural complexity of neuronal cells probably requires the expression of these two profilin isoforms [28]. Since, also astrocytes exhibit a ramified morphology and transcriptome analyses pointed to an additional expressed profilin isoform [45–47], we investigated the expression of PFN2a in astrocytes and addressed the functional relevance of PFN1 and PFN2a in astrocytic morphology and plasticity.

RNAi-mediated downregulation of either PFN1 or PFN2a, respectively, resulted in a significant decrease in the overall size of cultured astrocytes. This finding is consistent with the cell size reduction caused by the expression of an actin-binding deficient mutant of PFN1 [29] and the 15% reduction in process length of PFN1^-/-^ Bergmann glia [30]. Moreover, dissociated astrocytes expressing the shRNA specific for PFN2a exhibit a higher percentage of polygonal cells, indicating PFN2a as a regulator of astrocytic morphology. This result was supported by the analysis of astrocytes expressing the PFN2a specific shRNA in organotypic cortical slice cultures, which display a volume reduction. These findings suggest a decline in the amount of astrocytic processes and therefore complexity. Consistently, Michaelsen and colleagues [28] described PFN2a as an important modulator of dendritic architecture of CA1 pyramidal neurons.

In the present study, crucial function of both profilin isoforms was demonstrated concerning morphological plasticity, since the stellation observed in astrocytes upon forskolin treatment was abolished in the absence of profilins. Knockdown of either profilin isoform had a similar effect on stellation, which supports the idea of a functional compensation between the two profilin isoforms *in vivo* [24,33]. Forskolin is described as direct activator of the adenylate cyclase, thereby increasing the cellular amount of cAMP and subsequently activating PKA [48]. However, the application of Rp-cAMP as competitive PKA inhibitor was not sufficient to abolish stellation in cerebellar rat astrocytes [49]. Co-application of the cAMP raising with a competitive inhibitor may diminish its effectiveness [50], especially since cerebellar astrocytes are most susceptible to cAMP induced stellation [51]. Another recent study describes KT5720 as effective inhibitor of astrocytic stellation [52], which is in line with our results. The aforementioned study by Perez and colleagues [49] furthermore suggests of short time inactivation of PI3K upon forskolin treatment.

The inactivation of PI3K has various consequences on profilin phosphorylation states. First, it leads to a reduced amount of PFN directly phosphorylated by PI3K [53]. PI3K phosphorylated PFN was described to have an increased affinity to both G-actin and poly L-proline (PLP) without changes in the binding to phospholipids. Second, PKC downstream of PI3K is not activated, which in turn results in a reduced PFN phosphorylation [54]. Last, PI3K inactivation caused an inactivation of RhoA [49]. Its downstream effector ROCK thereby releases PFN causing a dephosphorylation of PFN [37]. ROCK-dependent phosphorylation of PFN at serine residue 137 abolished the interaction with PLP and reduced the affinity to G-actin [55]. With our study, we demonstrate the relevance of this PI3K dependent pathway for the phosphorylation of PFN1 in astrocytes, since we found a highly significant reduction of phosphorylated PFN1 upon forskolin-treatment. In contrast, the increase in the relative amount of phosphorylated PFN2a upon forskolin-treatment can most likely be attributed to PKA-dependent phosphorylation. Co-application of the PKA inhibitor KT5720 leads to a decrease in PFN2a phosphorylation compared to forskolin treated astrocytic cultures. Together, these data may indicate PFN2a as a phosphorylation target downstream of PKA and suggest an isoform-specific regulation of profilin isoforms via phosphorylation.

The alteration of the subcellular localization of endogenous profilin isoforms in response to forskolin-treatment or PKA inhibition suggests both profilins to accomplish functions in the nuclear compartment. Unspecific stimulation of dissociated neurons by KCl treatment resulted in an elevated amount of both profilin isoforms in the nuclei [27]. Interestingly, a more specific stimulation via BDNF application and thereby activation of the TrkB receptor pathway (for review [56]) exclusively led to a redistribution of PFN1 to the neuronal nucleus. Here, we found a PKA-independent, isoform-specific redistribution of PFN1 in astrocytes subjected to forskolin. This result suggests either a role for PFN1 downstream of PI3K, which would fit our results from the 2D gel electrophoresis or as nuclear downstream effector of the Epac signaling cascade (for review [57]). We found both profilin isoforms to translocate to the nucleoplasm upon PKA inhibition, indicating an overlapping nuclear function in that context. Stimulus-dependent isoform-specific translocation of profilins to the cellular nucleus therefore may represent a common mechanism to regulate nuclear actin dynamics [58]. The significance of PFN1 translocation to the cellular nucleus is yet not well understood. PFN1 interacts with nuclear actin [39] and co-localizes in the nucleus with several ligands like VASP or mDia1 [41]. Furthermore, PFN1 specific antibodies were shown to interfere with pre-mRNA splicing [40], which may point to a role of profilin in mRNA processing. Since cAMP elevation results in altered transcription mediated by both PKA and PKA-independent pathways [59], PFN1 translocation may present a novel molecular mechanism modifying transcription in this context. The application of the PKA inhibitor KT5720 led to an evident translocation of both PFN1 and PFN2a to the astrocytic nucleus. The formin mDia2, which interacts exclusively with PFN2a [28], was demonstrated to localize to cellular nuclei upon inhibition of the nuclear exporter CRM1 [60]. Simultaneous translocation of these two interaction partners may promote actin polymerization in the nucleus under certain circumstances.

In this study, the motility of astrocytic processes was found to be dependent on both PFN1 and PFN2a. Nevertheless, solely downregulation of PFN1 impaired actin dynamics in the astrocytic peripheral processes. These findings indicate differential, isoform-specific functions of profilin isoforms in regulating astrocytic protrusions. On the other hand, the reduction in dendritic spine numbers in CA1 pyramidal neurons caused by the RNAi-mediated downregulation of PFN2a was rescued by the expression of recombinant PFN1 [28]. In addition, other recent studies suggest a compensatory capacity of PFN1 and PFN2a in neuronal structure and function [24,33]. In line with this suggestion, the expression of an actin-binding deficient PFN1 had no consequences for the amount of astrocytic protrusions at basal conditions [29]. In our study, the astrocytes were cultivated in the absence of neurons, and consequently did not form tripartite synapses possibly resulting in different outcome as observed for star-like astrocytes *in situ*. Nevertheless, it was previously observed that the activity-induced motility of PAPs was drastically impaired by the expression of an actin-binding deficient PFN1 mutant [29]. These results are in line with the crucial function of PFN1 in actin dynamics and directed motility of astrocytic processes demonstrated in the present study. Remarkably, FRAP analysis of PFN1 overexpressing cancer cells also revealed impaired actin dynamics characterized by an elevated percentage of stable F-actin [61].

Around 60% of spines are contacted by dynamic astrocytic protrusions [62] continuously protruding and retracting in response to neuronal stimuli [6], thereby altering synaptic transmission [3]. Profilin-dependent changes in synaptic coverage by PAPs might as a consequence alter synaptic plasticity. As both profilin isoforms are linked to stimulus-dependent motility of astrocytic processes, a transduction of neuronal signals to the astrocytic cytoskeleton mediated by profilins is indicated.

**Fig 8:**
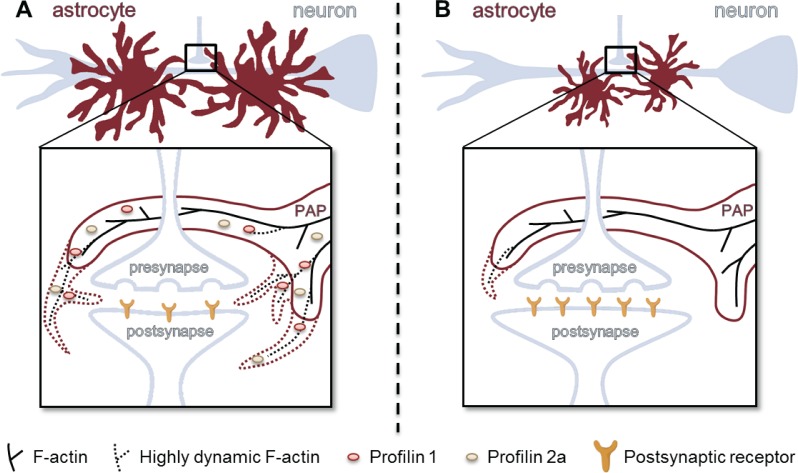
Illustration of the proposed cellular function of profilins in glia-neuronal networks. (A) In the presence of all actin binding proteins, astrocytes are very complex and occupy large areas. Their highly motile processes (PAP) sense and modify synaptic input on the engulfed synapse. Profilins might regulate the motility of these processes either by direct binding to actin (PFN1) or by influencing its dynamics (PFN2a). (B) The absence of profilins results in a reduced size and complexity of astrocytes. Motility of astrocytic processes deficient of profilin isoforms is drastically reduced. This may lead to altered synaptic properties and plasticity.

These new insights into the cellular functions of profilins in astrocytes are summarized in the model presented in Fig. 8. Here, astrocytes expressing both profilin isoforms show a complex morphology (Fig. 8A). Their small peripheral processes are highly dynamic, quickly responding to synaptic activity by actin cytoskeleton remodeling. Instrumental for this fast, actin-based dynamic of astrocytes are profilin isoforms. In contrast, profilin depleted astrocytes occupy a reduced area and are less complex (Fig. 8B). Moreover, their PAPs exhibit a drastically reduced motility, resulting in a delayed interaction with the respective synapse. This may be accompanied by an increased structural stability of the synapse [63]. Future prospects should include the role of astrocytic plasticity and its regulation by profilins to gain a comprehensive view on the interplay of astrocytes and neurons in neuronal transmission and synaptic plasticity.

## Materials and Methods

### Ethics statement

All animal experiments were performed in compliance with the German animal welfare law (TierSchG BGBl. S. 1105; 25.05.1998) and in strict accordance with the Directive 2010/63/EU of the European Parliament and the Council of the European Union of 22. September 2010. The mice were housed and handled in accordance with good animal practice as defined by FELASA. The protocol was approved by the responsible state office (Lower Saxony State Office of Consumer Protection and Food Safety) under permit number AZ 033.42502-05-A-002/08.

### Plasmids

PFN2a specific knockdown construct pRNAT-2.13-eGFP, was described elsewhere [28]. For PFN1 specific knockdown, the ds oligodeoxynucleotide GATCCCGTTGTTGATCAAACCACCGTGGTTGATATCCGCCACGGTGGTTTGATCAACAATTTTTTCCAAA was inserted into pRNATU6.3/Hygro (GenScript, Piscataway, USA), resulting in pRNAT-1.3-eGFP. pRNATU6.3/Hygro/siFLuc (GenScript) was used as control. Additionally, the reporter eGFP was replaced by mApple (Kind gift of Michael W. Davidson, National High Magnetic Field Laboratory, USA), resulting in pRNAT-1.3-mApple and pRNAT-2.13-mApple, respectively. Morphological analysis of astrocytes in slice cultures was performed with constructs where eGFP cDNA was exchanged with feGFP cDNA [28] to ensure precise detection of fine processes. For lentiviral constructs, shRNAs were cloned into the vector pRNAT U6.2/ lenti (GenScript). cGFP cDNA was exchanged for feGFP cDNA [28] and a WRPE site was included, resulting in pUsiFluc-GfW, pUsh1.3-GfW and pUsh2.13-GfW, respectively.

### Antibodies

For detection of PFN1, a polyclonal anti-PFN1 antibody (P7624, Sigma-Aldrich, USA) was used. PFN2a was detected with either a polyclonal anti-PFN2a antiserum [32] or a monoclonal anti-PFN2a 4H5 antibody [27]. Astrocytes were identified using monclonal anti-GFAP (G-A-5; Sigma-Aldrich) or polyclonal anti-GFAP (AB5804, Chemicon/Millipore, Billerica, USA) antibodies. As loading control for the Western blot an anti-GAPDH antibody (Acris, Herford, Germany) was used. Secondary antibodies conjugated with Cy2, Cy3, or Cy5 (Dianova, Hamburg, Germany) or HRP (Sigma-Aldrich) were used for visualization.

### Cell culture

Primary glia cell cultures were obtained from p5 mice as previously described [64] with modifications. Briefly, after decapitation, the cortices were isolated in ice cold Hank’s Balanced Salt Solution (HBSS, Life Technologies, Darmstadt, Germany). Subsequently, after removing all meninges the cortices were dissociated with both trypsin/EDTA for 8 min and mechanically with a fire-polished Pasteur pipette. After plating in DMEM with 10% FCS on uncoated tissue culture flasks (TPP, Trasadingen, Switzerland), cells were incubated at 10% CO_2_ and 37°C. Superficial oligodendrocytes and microglia were removed by shaking over night at DIV 7. The cultures were passaged at DIV 10–13 and plated on poly-L-ornithine (PLO, Sigma-Aldrich) coated glass cover slips or onto uncoated 10 cm TC dish (TPP). Medium was exchanged twice a week. Cortical organotypic slice cultures of postnatal day 5 mice (C57 Bl/6) were prepared as previously described [65].

### Cell transfection

Profilin levels in astrocytes were modulated by either transfection of expression plasmids or application of lentivirus coding for specific shRNAs. Transfection was performed using Lipofectamine2000 (Invitrogen, Carlsbad, USA) according to manufacturer’s advices either on DIV14 or DIV18, depending on the construct used [32]. After an incubation time of 4h, the transfection mix was replaced with DMEM containing FCS and the cultures were incubated until DIV 21. Organotypic cultures were transfected at DIV 7 using the Helios gene gun system (Biorad). Bullet preparation was performed using a ratio of 2:1 (milligrams of gold per microgram of DNA). Slices were transfected by shooting at a pressure of 100 psi through tissue culture inserts with a pore size of 3 μm to prevent gold clumps from damaging the cultures [28].

### Treatment of astrocytic cultures

To induce stellation in astrocytes, cells were serum starved for 2 hours followed by a 2 hours treatment with 10 μM forskolin (Sigma) in DMEM without FCS. For PKA-inhibtion, astrocytic cultures were incubated with 100 nM KT5720 (TOCRIS, Bristol, UK) for 4 hours under serum depletion.

### Production and administration of lentivirus

The lentiviral expression plasmids described above were cotransfected into HEK293T cells together with pCMV∆R8.91 [66] as packaging vector and pVSVG envelope vector for production of retroviral particles as described previously [67]. For transduction of primary glia cell cultures, 72h post transfection the supernatant was harvested, filtered and kept at −70°C. Briefly, cells were washed with PBS (0.137 mol/L NaCl, 0.0027 mol/L KCl, 0.0015 mol/L KH_2_PO_4_, 0.008 mol/L Na_2_HPO_4_; pH 7.3) and subsequently, new medium together with the lentiviral particles in a 1:2 dilution was added. Virus containing medium was exchanged 2–3 days after transduction. Cells were transduced with Usifluc-GfW or U2.13-GfW 9 days or with U1.3-GfW 5 days before the experiments. Expression visualized by GFP-fluorescence started 2 d after transduction.

### 2D gel electrophoresis and immunoblotting

PFN1 and PFN2a were detected in extracts of glia cell cultures which were harvested at DIV21 – 23. 20 μg of total protein was loaded onto SDS-PAA gels and blotted onto PVDF membranes.

For 2D gel electrophoresis, cells were lysed in a solution buffer containing 8 M urea and 2% CHAPS and subsequently, 50 μg of total protein was loaded onto Bluestrips (Serva, Heidelberg, Germany) for isoelectric focusing. Afterwards, the strips were placed onto SDS-PAA gels and blotted onto nitrocellulose membranes.

### Immunocytochemistry

Primary astrocytic cultures (DIV 21) were fixed with 4% formaldehyde in PBS, permeabilized with 0.2% Triton X-100 and subsequently stained with the appropriate primary and secondary antibodies. Cultures were counterstained with DAPI (4′,6-diamidino-2-phenylindole) (AppliChem, Darmstadt, Germany) washed and mounted with Fluoro-Gel (Emsdiasum, Hatfield, USA).

### Spreading assay

Spreading of transduced DIV14 primary astrocytes were analyzed by plating the cells on PLO coated coverslips. After 6 hours of incubation, cells were washed two times with PBS and fixed with 4% formaldehyde in PBS. Subsequently, they were stained for the astrocytic marker GFAP and for F-Actin with tetramethylisocyanate (TRITC) conjugated phalloidin. Using a 20x (1.25 NA) objective on an Axioplan 2 (Zeiss, Göttingen, Germany) fluorescence microscope. Astrocytes were classified into two groups: well-spread flat (group 1) and compact, round cells (group 2).

### Transwell assay

To analyze the motility of astrocytic processes, chemotactic attraction to FCS was used in a modified pseudopodium isolation assay [42,43]. Here, serum starved astrocytes were seeded in the upper compartment of a Boyden-chamber with a pore size of 3 μm (Greiner bio-one, Frickenhausen, Germany). DMEM containing 20% FCS in the bottom chamber was used to stimulate directed process outgrowth. Cells were allowed to spread their processes into the bottom chamber for 24 hours before being fixed and immunostained. Images of focal planes above and below the insert were taken with a confocal laser scanning microscope (LSM 510 Meta, Zeiss) using a multitrack technique to monitor the individual channels separately.

### Fluorescence recovery after photobleaching (FRAP):

For FRAP experiments, co-transfection of either the control plasmid, or sh-RNA expressing plasmid specific for PFN1 and PFN2a, respectively, with GFP-β-actin was performed. Controls and PFN2a knockdown plasmids were transfected 7 d ([28]) before the experiment, whereas PFN1 specific knockdown plasmid was transfected 3 d [32] before the experiment. All experiments were performed at DIV21 on small astrocytic processes. The cells were kept at 32°C in an open imaging chamber with a continuous perfusion (0.5 ml/min) of HBSS. The Olympus system BX61WI FluoView 1000 (FV1000) was used to excite GFP-β-actin with an excitation wavelength of 488 nm. The power of the excitation laser was adjusted to a low level (1–2%; 5–8 μW). Scan speed was set to 8 μs per pixel and a line averaging of two (Kalman filtering) were used for an image size of 640 × 128 pixels (final pixel size of 76nm) with a 60x water immersion objective (NA1.0). To increase the z-section’s depth the pinhole was opened to 500 μm and gain and offset values were set to zero. The photobleaching of a 4x4 μm area was performed using the SIM scanner unit Olympus FV5-LDPSU at an excitation wavelength of 405 nm and a power of 100% (8 mW) for 500 ms. Baseline was determined by taking 5 images at two second intervals before bleaching. The fluorescence recovery was monitored for 3 min after bleaching.

Fluorescence recovery was quantified using the Olympus software FV1000. Background fluorescence was measured in a random 4x4 μm area without astrocytic processes. The average relative intensity of the region of interest (ROI) was calculated and corrected by a double normalization [68]. The turnover time (half-time of recovery τ_1/2_) was calculated as the time in seconds at which the fluorescence intensity reached half of its maximum pre-bleaching value. Different actin fractions were calculated based on the exponentially fitted recovery curves using the equation $F(t)=1-fs-ffe^{-\frac{t}{\lambda }}$ (with λ as time constant of a single exponential function describing the recovery after photobleaching) [44] in GraphPad Prism 5. Here, an ‘immobile’ F-actin fraction stably associated and not recovering during the time of observation (*fs*) and a ‘mobile’, dynamic F-actin fraction (*ff*), which recovers during the experiment, are defined.

### Image analysis

To analyze the subcellular localization of PFN isoforms, DIV 21 primary astrocytic cultures were fixed and immunostained. Six images per experiment were taken with the Olympus System BX61WI FluoView 1000 equipped with a 40x oil immersion objective (NA 1.3). Fluorescence intensity of the profilin staining was measured by FIJI software [69]. The ratio between one area co-localized with DAPI staining and one area of the same size beneath the DAPI staining was used for analysis.

Knockdown efficiency was determined in dissociated cultures transfected as earlier mentioned by confocal microscopy with the Olympus System BX61WI FluoView 1000 equipped with a 40x oil immersion objective (NA 1.3). Transfected and GFAP-positive cells were selected, imaged and the fluorescence intensity of the PFN1 or PFN2a staining in areas excluding of the nucleus of transfected versus non-transfected cells from the same image were compared using FIJI software. The resulting ratio was used to quantify the effect of the respective knockdown construct in astrocytes.

Morphological analysis of astrocytes was performed using a 63x (1.4 NA) objective on an Axioplan 2 (Zeiss, Göttingen, Germany) fluorescence microscope.

For morphological analysis in dissociated cultures, astrocytes were categorized into polygonal and stellate astrocytes by the ratio of cell perimeter to cell area. Transfected, vital and GFAP positive cells were selected and imaged with an Axioplan 2 fluorescence microscope (Zeiss, Germany) equipped with a 63 x objective (1.32 NA). Cell perimeter and cell area were measured with FIJI software. Astrocytes with a ratio > 0.25 were totaled as stellate. For the analysis of astrocytes from organotypic cultures, slices were fixed at DIV14. Volume analysis was performed using Bitplane Imaris 7.0. Here, a virtual surface was created with the respective wizard setting surface area detail level at 0.2 μm and the background subtraction at 0.8 μm. The resulting volume data were exported to Microsoft Excel and used for analysis.

### Statistical analysis

The statistical analysis was performed using Microsoft Excel and GraphPad Prism. The data obtained were compared between two different experimental conditions using an unpaired two-tailed Student’s t-test. For comparison of more than 2 different groups a one-way ANOVA or a two-way ANOVA followed by a *post-hoc* Tukey’s Multiple Comparison Test was used. Values of p<0.05 were considered as being significant and plotted as follows *p<0.05; **p<0.01; ***p<0.001. Data shown are indicated as mean ± SEM.

## Supporting Information

S1 Fig shRNA mediated knockdown of profilin isoforms. (A) Lentiviral vector construct targeting PFN1 or PFN2a mRNA (“shRNA”) specifically and expressing eGFP-f under the control of a CMV promoter. (B) Western blot analysis of astrocytic cultures transduced with the indicated lentiviruses. Administration of PFN1 specific lentivirus (Ush1.3) led to a reduced amount of PFN1 in the culture, whereas the amount of PFN2a was not affected. On the other hand, the application of the PFN2a specific lentivirus (Ush2.13) resulted in a reduction of PFN2a in the cells, whereas the level of PFN1 was not reduced. GAPDH was used as loading control. (B’) Quantification of the relative, GAPDH normalized amount of PFN1 in Ush1.3 transduced cultures (31.3%) in comparison to UsiFluc transduced cultures. (B”) Quantification of the relative, GAPDH normalized amount of PFN2a in Ush2.13 transduced cultures (49.6%) in comparison to UsiFluc transduced cultures. (C) Vector construct targeting PFN1 or PFN2a mRNA (“shRNA”) specifically and expressing mApple under the control of a truncated CMV promoter. (D) Statistical analysis of fluorescence intensities of transfected astrocytes. Ratios between transfected and non-transfected cells were used. The mean fluorescence intensity ratio of non-transfected astrocytes stained with the respective antibody was set to 1. Analysis of astrocytes transfected with either pRNAT-1.3 or pRNAT-2.13 exhibits a highly significant reduction to 0.51±0.04 or 0.53±0.03, respectively. Quantitative data obtained from 4 independent experiments was tested for significance by unpaired two-tailed Students’ t-test to compare between two different experimental conditions. Significance is indicated as follows *p<0.05; **p<0.01; ***p<0.001. Data are shown as mean ± SEM.
(TIF)

S2 Fig Knockdown of profilin isoforms resulted in a reduced cell size. Cell perimeter and area of transfected astrocytes were measured by use of the software FIJI. (A) Cell perimeter of PFN1 (391.9±29.6 μm) or PFN2a (385.9±24.8 μm) knockdown astrocytes was significantly reduced compared to MOCK transfected cells (538.0±38.0 μm). (B) Cell area of both PFN1 (1928±163 μm²) and PFN2a (1938±147 μm²) knockdown cells are significantly reduced compared to MOCK transfected cells (2513±191 μm²). Quantitative data obtained from 4 independent experiments was tested for significance by unpaired two-tailed Students’ t-test to compare between two different experimental conditions. Significance is indicated as follows *p<0.05; **p<0.01; ***p<0.001. Data are shown as mean ± SEM.
(TIF)

S3 FigFluorescence recovery after photobleaching analysis of small astrocytic processes.A square of 4x4 μm was bleached from astrocytes co-expressing eGFP-actin and siFluc (MOCK, 17 cells). Turnover time of the actin filament is indicated by τ_1/2_, which gives a qualitative measurement of the recovery speed at constant experimental conditions and correspondents to the time point were 50% of the fluorescence is recovered. The presented curve was obtained by fitting the data using the equation for fluorescence recovery after photobleaching $F(t):F(t)=1-fs-ffe^{-\frac{t}{\lambda }}$ as described [44]. Actin pools gained from the aforementioned curve fitting are indicated as stable (*fs*, black) and dynamic (*ff*, red).(TIF)
