# Supplementary figures and images for: Profilin Isoforms Modulate Astrocytic Morphology and the Motility of Astrocytic Processes
Source: PLoS One. 2015 Jan 28;10(1):e0117244. doi: 10.1371/journal.pone.0117244 (PMC4309604; doi:10.1371/journal.pone.0117244)

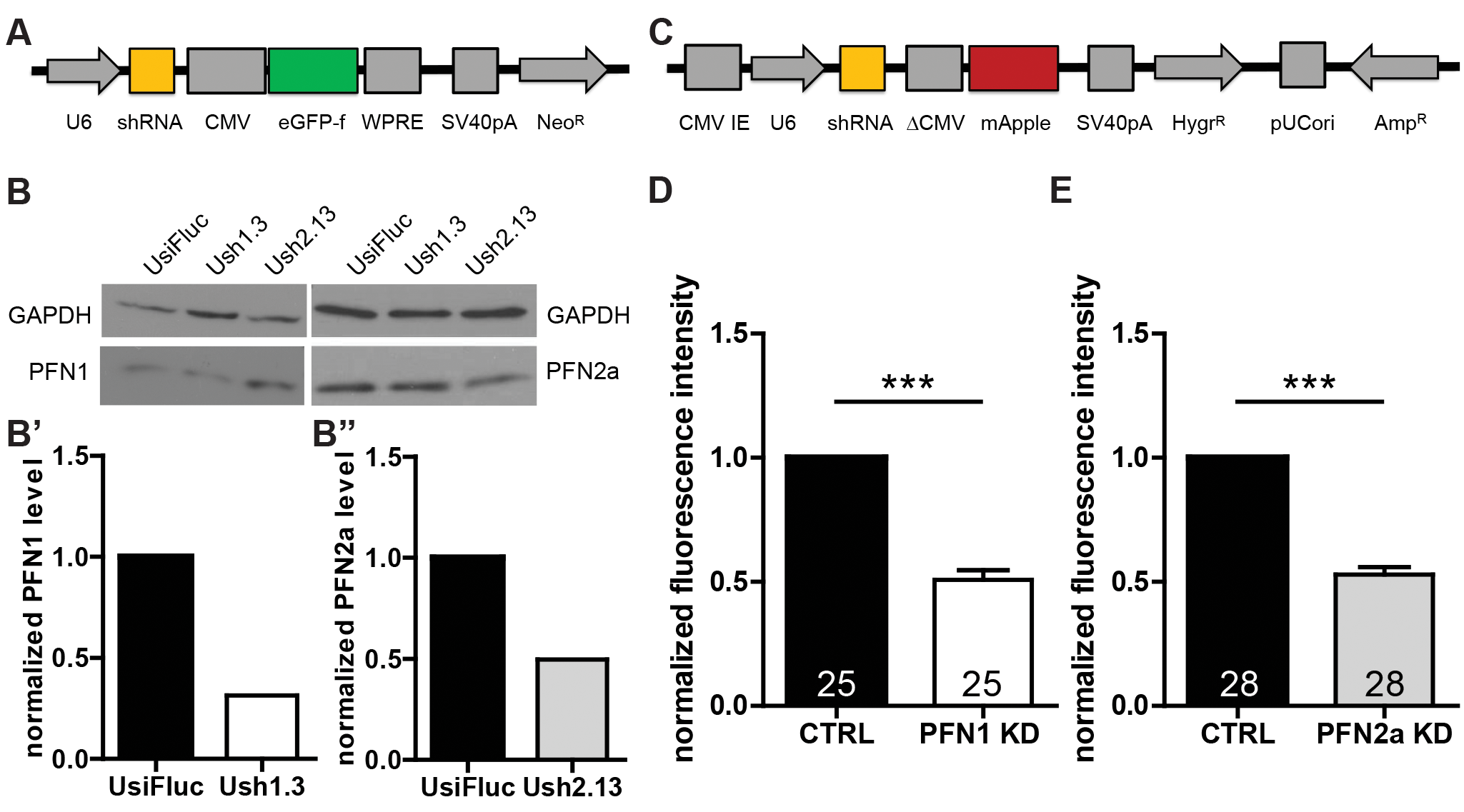

Supplement: S1 Fig — (A) Lentiviral vector construct targeting PFN1 or PFN2a mRNA (“shRNA”) specifically and expressing eGFP-f under the control of a CMV promoter. (B) Western blot analysis of astrocytic cultures transduced with the indicated lentiviruses. Administration of PFN1 specific lentivirus (Ush1.3) led to a reduced amount of PFN1 in the culture, whereas the amount of PFN2a was not affected. On the other hand, the application of the PFN2a specific lentivirus (Ush2.13) resulted in a reduction of PFN2a in the cells, whereas the level of PFN1 was not reduced. GAPDH was used as loading control. (B’) Quantification of the relative, GAPDH normalized amount of PFN1 in Ush1.3 transduced cultures (31.3%) in comparison to UsiFluc transduced cultures. (B”) Quantification of the relative, GAPDH normalized amount of PFN2a in Ush2.13 transduced cultures (49.6%) in comparison to UsiFluc transduced cultures. (C) Vector construct targeting PFN1 or PFN2a mRNA (“shRNA”) specifically and expressing mApple under the control of a truncated CMV promoter. (D) Statistical analysis of fluorescence intensities of transfected astrocytes. Ratios between transfected and non-transfected cells were used. The mean fluorescence intensity ratio of non-transfected astrocytes stained with the respective antibody was set to 1. Analysis of astrocytes transfected with either pRNAT-1.3 or pRNAT-2.13 exhibits a highly significant reduction to 0.51±0.04 or 0.53±0.03, respectively. Quantitative data obtained from 4 independent experiments was tested for significance by unpaired two-tailed Students’ t-test to compare between two different experimental conditions. Significance is indicated as follows *p<0.05; **p<0.01; ***p<0.001. Data are shown as mean ± SEM. (TIF) [file pone.0117244.s001.tif]

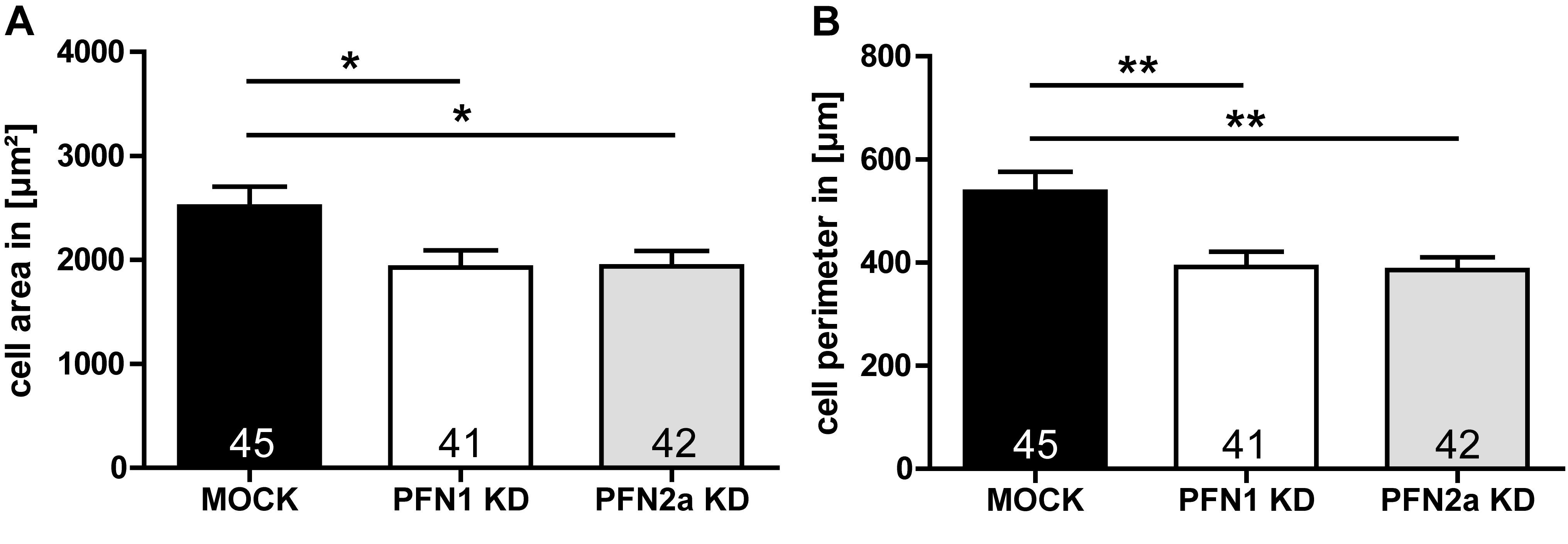

Supplement: S2 Fig — Cell perimeter and area of transfected astrocytes were measured by use of the software FIJI. (A) Cell perimeter of PFN1 (391.9±29.6 μm) or PFN2a (385.9±24.8 μm) knockdown astrocytes was significantly reduced compared to MOCK transfected cells (538.0±38.0 μm). (B) Cell area of both PFN1 (1928±163 μm²) and PFN2a (1938±147 μm²) knockdown cells are significantly reduced compared to MOCK transfected cells (2513±191 μm²). Quantitative data obtained from 4 independent experiments was tested for significance by unpaired two-tailed Students’ t-test to compare between two different experimental conditions. Significance is indicated as follows *p<0.05; **p<0.01; ***p<0.001. Data are shown as mean ± SEM. (TIF) [file pone.0117244.s002.tif]

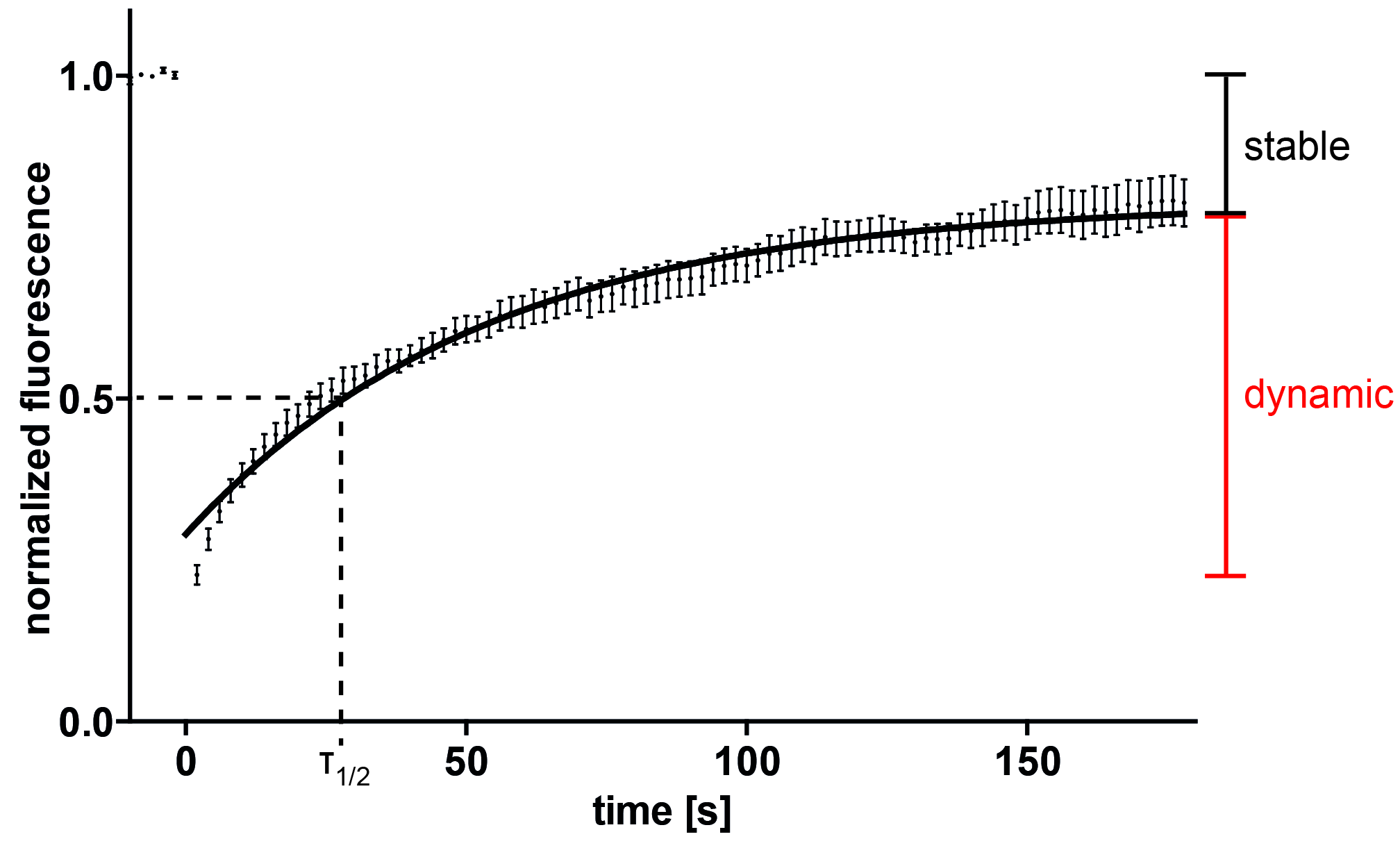

Supplement: S3 Fig — A square of 4x4 μm was bleached from astrocytes co-expressing eGFP-actin and siFluc (MOCK, 17 cells). Turnover time of the actin filament is indicated by τ1/2, which gives a qualitative measurement of the recovery speed at constant experimental conditions and correspondents to the time point were 50% of the fluorescence is recovered. The presented curve was obtained by fitting the data using the equation for fluorescence recovery after photobleaching F(t):F(t)=1−fs−ffe−tλ as described [44]. Actin pools gained from the aforementioned curve fitting are indicated as stable (fs, black) and dynamic (ff, red). (TIF) [file pone.0117244.s003.tif]
